# Towards a rights-based approach for disabled women’s access to abortion

**DOI:** 10.1093/medlaw/fwae026

**Published:** 2024-07-17

**Authors:** Magdalena Furgalska, Fiona de Londras

**Affiliations:** York Law School, University of York, York, UK; Birmingham Law School, University of Birmingham, Birmingham, UK

**Keywords:** abortion, CRPD, disability, human rights, pregnant women, reproductive agency

## Abstract

This article adds to the still limited scholarship on the impact of abortion laws and policies on people with disabilities and those with diminished capacity who seek abortion. We argue that neither the legal nor policy framework currently operating in England and Wales adequately incorporates and protects the rights of people with disabilities or those experiencing mental ill-health. Rather, the law and policy framework jeopardizes their reproductive agency. We argue that greater attention to and incorporation of standards contained within the UN Convention on the Rights of Persons with Disabilities (including the sources produced by its Committee) and implementation of guidelines produced by the World Health Organization would result in a rights-affirming framework that supports disabled women’s reproductive agency, enhances their effective enjoyment of human rights, and supports them in accessing quality abortion care.

## I. INTRODUCTION

The UN Convention on the Rights of Persons with Disabilities (CRPD) calls for the provision of adequate support to people with disabilities (including people with mental illness) to ensure that they can enjoy and exercise their rights on an equal basis with others. Although this has clear resonance for the rights of disabled women^1^ seeking access to abortion, there has been limited work on the CRPD’s implications in that regard. Apart from maintaining that pregnant disabled women should not be subject to forced abortions or stigmatized for seeking abortion care,^2^ the relevant human rights bodies have not engaged in discussions about the role of the CRPD in ensuring pregnant women and girls are enabled to access quality abortion care. This same absence is notable in much of the scholarly literature.^3^ Indeed, as the World Health Organization (WHO) has noted, the impact of abortion laws and policies on disabled women and people with diminished capacity remains under-researched.^4^ To begin to address this gap, we focus in this article on the burdens experienced by women with mental or cognitive disabilities in seeking access to abortion care. Within clinical and health systems provision and planning, disabled women who seek abortion are considered ‘complex’ cases. While experiences, of course, differ across disabled people^5^ as they do across all people, such complexity is attributed to challenges for disabled women in managing contraception, heightened risks in sexual behaviour, vulnerability to sexual exploitation,^6^ and reduced awareness of the signs of pregnancy experienced by some disabled people.^7^

To date, however, discussion about the relationship between abortion and disability has been dominated by concerns about the stigmatizing effects of disability and foetal anomaly^8^ being listed as ‘grounds’^9^ for access to lawful abortion. In some cases, these concerns have been mobilized in an attempt to reshape abortion-related human rights discourse to centre the foetus.^10^ Some have sought to use the CRPD as a ground to argue that the ‘disabled foetus’ has rights under the Convention,^11^ with obvious implications for the permissibility of abortion. Through these arguments, advocates seek to establish the foetus, diagnosed with a foetal anomaly, as a rights-bearer under the CRPD. They argue that abortion in such circumstances violates these purported foetal rights and, in some cases, advocate for the prohibition of prenatal testing that may identify foetal abnormalities.^12^

In 2018, the Committee on the Elimination of Violence against Women and the Committee on the Rights of People with Disabilities jointly expressed concern

at the increasing rollback and regression on respect for international human rights norms that threaten sexual and reproductive health and rights of women, including women with disabilities, who continue to experience intersecting forms of discrimination^13^

that such discourse reflects. Although anti-abortion arguments rooted in purported foetal rights are manifestly inconsistent with the well-established principle that internationally protected human rights accrue at birth, they point to the policy complexities of respecting pregnant people’s sexual and reproductive health while fulfilling states’ CRPD obligations to raise societal awareness about people with disabilities with an aim to foster respect for their rights and dignity and also to combat negative stereotypes and prejudices that exist within the societies.^14^

Abortion is highly regulated in England and Wales. The primary legislation, the Abortion Act 1967, regulates abortion through the paradigm of criminal law,^15^ with exceptions shaped by grounds, gestational limits, and multi-doctor certifications,^16^ and provision subject to conscientious objection.^17^ Up to 24 weeks gestation abortion is legally available where continuation of the pregnancy would pose a greater risk to the physical or mental health of the pregnant woman or her existing children than termination.^18^ Beyond the 24-week limit, abortion is available if there is a risk of grave permanent injury to the pregnant women,^19^ if the continuation of pregnancy poses a risk to the pregnant person’s life,^20^ or if there is a ‘substantial risk’ of severe foetal impairment.^21^

Within this context, we seek to reorient dominant debates about the relationship between abortion and disability by focusing on whether existing law and policy frameworks for accessing abortion in England and Wales are consistent with disabled women’s abortion-related rights. Abortion law and policy shape the pregnancy-related experiences and care of disabled women who are pregnant just as they do of all pregnant people, including those who do not seek abortion. We show that existing frameworks of law and policy are not sufficient to ensure disabled women’s rights are respected, protected, and enjoyed. Drawing on international human rights law, we argue that the CRPD provides a way to reform abortion law and policy to support disabled women in making abortion-related decisions and ensure that abortion is accessible for people with disabilities.

The article proceeds as follows. First, we illustrate that there is a substantial body of international human rights law, and rights- and evidence-based guidance on abortion law and policy that provides clear guidance on how abortion law might be constructed to maximize rights compliance for all pregnant people who seek it. Drawing on both international human rights law and the WHO’s *Abortion Care Guideline*, we argue that effective enjoyment of disabled women’s rights requires law and policy frameworks that support their access to quality abortion care. Building on the earlier work of Piers Gooding, we argue that CRPD-specific rights provide useful building blocks^22^ for creating a revised law and policy framework that would support disabled women’s access to quality abortion care. Second, we consider the Abortion Act 1967 in light of these international legal and policy standards, demonstrating significant human rights shortfalls of the existing law. In particular, we identify human rights shortfalls relating to (i) the provision of consent and supported decision-making for disabled abortion seekers, (ii) the persistence of gestational limits in abortion law, and (iii) the structure of service provision in abortion care in England and Wales. Overall, we argue that the established and foreseeable challenges of abortion access for disabled women (and provision for those who provide abortion care) reveal the need for further research into the everyday realities of abortion seeking for disabled women, girls, and pregnant people in England and Wales and the development of a new, rights- and evidence-based approach to supporting disabled women within abortion care provision.

## II. REPRODUCTIVE AGENCY IN HUMAN RIGHTS LAW: EVIDENCE-BASED LAW-MAKING

Although it is often said that there is no international human right to abortion, close analysis of the international human rights law corpus reveals a substantial body of law outlining the situations in which people are entitled to access abortion, and states’ obligations to ensure abortion is available, accessible, acceptable, and of good quality.^23^ The obligation to prevent and reduce maternal mortality and morbidity^24^ is well established and requires steps to ensure that pregnant women are not forced to opt for unsafe abortion,^25^ and that states reconsider their laws, including laws relating to abortion.^26^ Laws that prohibit or make inaccessible healthcare that only women need are considered discriminatory.^27^ International human rights bodies increasingly call on states to decriminalize abortion, recognizing the human rights harms that are associated with criminal regulation of reproductive healthcare.^28^ International human rights law does not reject grounds-based approaches to abortion regulation, but General Comment No 36 of the UN Human Rights Committee provides that

[a]lthough States parties may adopt measures designed to regulate voluntary termination of pregnancy, those measures must not result in violation of the right to life of a pregnant woman or girl, or her other rights under the Covenant.^29^

Thus, to satisfy women’s right to life, abortion must be available in very wide circumstances indeed.

It is now clear that a rights-based approach to abortion requires more than making abortion legal and extends to questions of the availability and accessibility of abortion, reinforcing the need to eliminate everyday barriers to abortion care to protect the health and reproductive choices of pregnant people. The most significant expression and demonstration of the value of this approach to date can be found in the WHO’s *Abortion Care Guideline*,^30^ which proposed seven recommendations for abortion law and policy that reflect human rights standards, clinical evidence, and quantitative and qualitative evidence of abortion-related outcomes and provision. These recommendations include the full decriminalization of abortion,^31^ provision of abortion on request^32^ and a move away from gestational age limits.^33^ In addition, the *Guideline* addresses policy-related regulations that make abortion inaccessible and unavailable in practice, such as abortion care pathways requiring referrals, third-party authorizations, and mandatory waiting periods, highlighting the damaging nature of such measures and interference with women’s and girls’ human rights.^34^ Reflecting an extensive review of available evidence, these recommendations reveal the disjuncture between paradigmatic approaches to abortion regulation, health outcomes, and human rights protection. They thus point to the rights-related challenges posed to all women by the Abortion Act 1967, challenges that are exacerbated for women with disabilities.

As recognized by the WHO, there is scant knowledge of disabled people’s experiences of accessing abortion care and how they are supported to do so.^35^ This is surprising, not only because of the particular challenges of accessing and providing high-quality abortion care to people with disabilities but also because research suggests that the most common reproductive decision of women with psychiatric illness is about termination of pregnancy.^36^ Furthermore, a recent systematic review suggests that women with mental ill-health are rarely incapacitous to decide about termination, although those with more serious psychiatric diagnoses like schizophrenia were commonly considered not to have capacity to decide about termination.^37^ The studies reviewed in that paper suggested—as our analysis below will also do—that time-sensitivity, the complex and unpredictable course of psychiatric illness, and the involvement of many stakeholders in decision-making and care provision meant that pregnant women’s complex needs were not met in decision-making processes or required significant resources and processes to be already in place, such as availability of multidisciplinary teams for management of abortion care, advance care planning, and careful use of surrogate decision-making.^38^ Combined with our analysis below, this indicates that there is a close yet unexplored intersection between disabled women’s reproductive agency and legal and service provision frameworks for abortion, especially in light of the English legal regime. In particular, it suggests that these law, policy, and service provision frameworks are not adequate to ensure that pregnant women with disabilities, including people experiencing mental ill-health, receive appropriate, supportive, rights-based, and health-maximizing abortion care.

We argue that all of these frameworks can be improved through serious engagement with international human rights law, including the CRPD. The WHO, which has the fulfilment of the right to health as part of its constitutional mandate, has long sought to integrate human rights with clinical and service-level evidence and insight in developing guidelines for health care provision. This commitment is reflected in both the 2022 *Abortion Care Guideline* and other, generally applicable, guidance produced by the Organization to support health and rights maximization for all, including people with disabilities. In 2023, it published *Mental health, human rights and legislation: guidance and practice* to articulate and guide reform of mental health laws based on rights-based approaches, centred on ‘respect for legal capacity and free and informed consent, without discrimination’.^39^ This publication builds on the work of QualityRights, an initiative to improve access to good quality social care and mental health services established in 2012.^40^ Together, these interventions seek to further implement CRPD rights, and thus add to the general recommendations in the *Abortion Care Guideline* to suggest a pathway for doing so in the context of abortion law and policy.

In spite of this, and notwithstanding its clear potential to underpin meaningful, agentic, and rights-based access to abortion for pregnant people with disabilities, the CRPD has not yet been applied to its fullest potential in relation to abortion. Instead, as mentioned Section I, much of the debate to date has centred around what the CRPD might have to say regarding ‘disability-selective’ abortions. Given the content of the Abortion Act 1967, the Committee on the Rights of Persons with Disabilities has raised questions concerning this in its Concluding Observations on the United Kingdom and Northern Ireland. In its 2017 Concluding Observations, it stated:

12. The Committee is concerned about perceptions in society that stigmatise persons with disabilities as living a life of less value than that of others and about the termination of pregnancy at any stage on the basis of fetal impairment.

13. The Committee recommends that the State party amend its abortion law accordingly. Women’s rights to reproductive and sexual autonomy should be respected without legalizing selective abortion on the grounds of fetal deficiency.^41^

Despite some anti-abortion campaigners’ interpretation of such concerns as pointing towards a need to limit access to abortion,^42^ when reading both paragraphs together, it is quite clear that the Committee’s concerns are best met by facilitating abortion on request and moving away from a grounds-based approach. Indeed, across the board, the evidence suggests that providing abortion on request is ‘the most effective approach to ensuring that abortion is available in the circumstances required by’^43^ international human rights law. Taking seriously the intention of the CRPD, namely to promote the rights of women with disabilities, which requires specific attention and efforts to protect their equal reproductive rights, points towards not only revising elements of the Abortion Act 1967 that tend to limit these rights (including grounds-based approaches and gestational age limits), but also taking positive steps to support access to abortion for disabled pregnant people who choose it.

To strike a fair balance between autonomy and protection for those considered more vulnerable in everyday life, the CRPD places particular emphasis on human diversity, equality of legal capacity, and human rights as tools to protect one’s ability to pursue one’s wishes and preferences. Thus, it requires that persons with disability have the right to recognition everywhere as persons before the law,^44^ and that states ensure persons with disabilities can exercise legal capacity (an ability to hold and exercise rights) on an equal basis with others^45^ which may necessitate the provision of additional support measures.^46^ Such measures should be appropriately safeguarded, proportionate, and tailored to the individual’s circumstances.^47^ This Convention thus aims to protect people with disabilities from violations of their bodily and mental integrity^48^ by emphasizing the need to empower people to exercise choice and self-determination. This is clearly coherent with the role of the right to informed consent within international human rights law, including the right to the highest attainable standards of physical and mental health.^49^

Article 1 of the Convention states that the very ‘purpose of the present Convention is to promote, protect, and ensure the full and equal enjoyment of all human rights and fundamental freedoms by all persons with disabilities, and to promote respect for their inherent dignity’. The right to equal recognition before the law and equal rights to exercise one’s legal capacity clearly indicate and seek to ensure the full realization of the (obvious) proposition that people with disabilities hold all the same rights as those who are not disabled. This includes international human rights relating to access to abortion. The major contribution of the CRPD is to recognize that people with disabilities may need access to supported decision-making to exercise their legal capacity on an equal basis with others, and indeed obliges states to provide for that.

As a concept, ‘supported decision-making’ refers ‘to a collection of various demands rather than having a single fixed meaning’,^50^ but at a base level it describes promoting ‘autonomy with support’^51^ so that people with disabilities can make decisions that are in line with their own sense of self and their values and can live their lives as they wish to. Thus, supported decision-making should not be understood narrowly as referring to decision-making processes only; it is more accurate to see it as measures and efforts to achieve the full range and enjoyment of human rights.^52^ In reality, supported decision-making can find many expressions, from formal mechanisms like advance directives to using simple language or other means of communication to enable people to gain an understanding of the world around them and provide them with opportunities to do or have things that are important to them. The gap identified by the WHO in relation to the impact of laws and policies on pregnant people with disabilities is much wider when considered through the CRPD lens; the general limited interest to date in this area leads to a lack of robust evidence about how pregnant people with disabilities who do not wish to progress with their pregnancy may be supported in pursuing their choice to access abortion and provided with meaningful and specialized access to quality abortion care. It suggests a need to develop new, evidence-based, rights-affirming pathways of support.

The CRPD itself indicates how this might be achieved. For example, the CRPD principle and obligation of accessibility^53^ place a responsibility on state parties to ensure that the provision of information is accessible and removes barriers to equal access to such information, as well as in physical spaces. The CRPD makes accessibility both an explicit norm and a right in ways that are not present in other sources of international human rights laws.^54^ The idea of accessibility in relation to abortion is important in supporting disabled pregnant women; the provision of information and physical environments where abortion occurs can arguably have a profound impact on women’s well-being and their legal capacity. The right to freedom of expression contained in Article 21 places a positive duty on the state to provide information in formats that are appropriate for disabled people, thereby widening the means by which information is provided, including on the Internet. The importance of Article 21 rights in relation to supporting pregnant disabled people in accessing abortion is striking: accessible information promotes meaningful reproductive decision-making and minimizes the chances of reproductive coercion (including forced abortion) from taking place.

The right to health without discrimination protected in Article 25, places an obligation on states to:

[p]rovide persons with disabilities with the same range, quality and standard of free or affordable health care and programmes as provided to other persons, including in the area of sexual and reproductive health and population-based public health programmes

as well as provide services that allow for early detection and identification of health needs to ensure appropriate interventions are given in a timely manner and ensure that people with disabilities can exercise free and informed consent. Finally, Article 6 obliges the states parties to take specific measures to ensure women with disabilities have full and equal enjoyment of all human rights. The specific provisions of the right to health are clearly significant when it comes to accessing abortion care, especially when modes of regulation include restrictive grounds, criminalization, and restrictions in terms of access. Nonetheless, when one considers the wide range of relevant rights in the CRPD as building blocks relevant to abortion care, the need for a framework to support decision-making in terms of both continuation of pregnancy and, where termination is chosen, access to and methods of abortion care, becomes axiomatic.

## III. ‘COMPLEX CASES’ AND HUMAN RIGHTS SHORTFALLS

Our previous work has shown that the core elements of abortion law in this jurisdiction—gestational limits, grounds-based approaches,^55^ criminalization,^56^ and third-party authorization–have negative impacts on both health and non-health-related outcomes, and have particular implications for people with disabilities or reduced mental capacity.^57^ These established difficulties within the legal framework are further exacerbated by the broader frameworks of medical law and service provision in England and Wales.

### A. Gestational limits and decision-making capacity

First, as already mentioned, the Abortion Act 1967 imposes gestational limits on access to abortion. While gestational limits can act as barriers to abortion access for all pregnant people, it is established that providing specialized abortion care to pregnant women, they can be especially challenging for disabled women.^58^ This is because disabled women ‘may experience difficulties sensing and interpreting their bodies’ signals and may also ‘be challenged in communicating these bodily experiences to others in time’.^59^

A case reported by Jean O’Hara^60^ in 1989 illustrates the challenges posed by gestational limits and ‘improvising’ best practice in complex abortion cases.^61^ O’Hara’s report discusses a case of an intellectually disabled woman in a London hospital whose IQ was deemed to be below 40, placing her in the legal category, then known as ‘severely mentally handicapped’. Her pregnancy was discovered at 24 weeks, placing her close to the gestational age limit for legal abortion. There were clear concerns about how she became pregnant while in a hospital, and it was later found that she had been sexually abused for many years. O’Hara reflects that ‘no one seemed to know how to best handle the situation’^62^ and that the hospital’s ‘legal advisers were also extremely uneasy with the whole situation and were unable to give us clear guidelines upon which to base our recommendation’.^63^ The decision on how to proceed fell on treating doctors who, at the time, were not able to find any guidelines on supporting or navigating a decision-making process in a scenario with high levels of complexity involving disability, time sensitivity, legal restrictions, the history of sexual abuse, and the need to provide meaningful emotional support for the pregnant person. Ultimately, concerned about gestational duration and the permissibility of abortion (especially given the lack of meaningful legal advice available to them), the treating clinicians abandoned the possibility of abortion and opted for continuation of pregnancy and adoption of the child at birth.

Having decided to proceed with the pregnancy and adoption, the treating clinicians felt it was necessary for the patient to develop some understanding of the situation. Initially, the approach adopted by the medical team consisted of using simple and basic language to explain to the patient that she had become pregnant.^64^ This resulted in slow progress and prompted the team to communicate with the aid of pictures as well as ‘anatomically correct dolls’^65^ to deepen the patient’s understanding of what was happening with her body. They later began to introduce the concept of adoption explaining that once born, the child would be given away to ‘loving parents’.^66^ At this point, O’Hara reported that with time, the patient gained ‘a remarkable understanding’ from initially showing ‘ambivalence to the loss of her future child to later actually crying’.^67^ The pregnant person’s growing understanding of what was happening prompted the medical team to continue an ‘intense level of emotional support’,^68^ which included supporting the bereavement process by producing ‘a storyboard of her own pregnancy, adding to it photographs at different stages, and completing it with the birth and adoption’.^69^ Prior to pregnancy, the patient suffered from intense hair-pulling behaviour, which lessened during pregnancy as the staff adopted a compassionate approach focused on giving the patient compliments, bringing her bright-coloured maternity clothing, and allowing her to choose ones she liked, getting her make-up and perfume to wear and introducing her to a music therapist who helped her explore and understand her emotions. The hospital provided the pregnant woman with a private room, nicely and newly decorated, to prevent her from feeling abandoned or punished. Ultimately, the pregnancy concluded and the baby was, indeed, adopted. There is no evidence that the preferences of the pregnant person were formally factored in the decision-making about continuation of pregnancy or the future care of her child. Indeed, the case note in question largely reflects on the clinician’s experience of seeking to provide care in the pregnant woman’s best interest in this case. Reflecting on her own role and the role of other professionals, O’Hara stated:

there are no clear rights or wrongs in dealing with such a complex situation. During the months preceding the birth there were numerous hurdles to overcome, causing us to question our own morals, stretching our skills and therapeutic techniques and testing the practicalities of working in a truly multidisciplinary settings which involved liaison between departments, hospitals and health and local authorities. The anxieties engendered in us all had to be recognised and supported.^70^

The work of O’Hara and her team in supporting the patient’s understanding and emotional well-being represents a meaningful example of support, although that cannot mitigate the broader human rights implications of living in an institutionalized care setting where the woman in question was clearly at risk of sexually abusive behaviour or, indeed, the fact that decisions were clearly made *for* her rather than *with* her. Even after acknowledging the commitment to care shown to the patient during pregnancy, it is very clearly the case that supporting the patient’s reproductive agency was not given much consideration. Having failed to enable the pregnant person to understand the situation she was in and support her decision-making in a timely manner, the woman in question encountered the gestational limit for access to abortion so that she could not meaningfully decide whether to continue her pregnancy. While the treating team supported the patient’s understanding of pregnancy and adoption, the same level of support could have been provided to ascertain whether the woman in question wanted to terminate the pregnancy. However, the patient in this case was not supported to do that partly, though not completely, because of the temporal pressure of the gestational age limit. Although more than 30 years old, this report of medics’ attempts to support a woman with intellectual disability highlights several conundrums that persist in contemporary practice.

The first of these relates to supporting disabled women in reaching decisions about whether to continue a pregnancy. As the case just recalled illustrates, at least some women with disabilities effectively have this decision made for them either because of assumptions about their disability, their ability to continue with pregnancy, or—as suggested by the above case—clinician assumptions that continuation of pregnancy would compound existing distress. O’Hara’s account suggests that she formed the view that abortion was preferable because of the background of sexual abuse leading to pregnancy, her assumptions about whether women *generally* wish to continue pregnancy in such circumstances, and her concerns for the pregnant woman’s mental wellbeing, It is clear from the report that treating doctors considered abortion carefully and thought that it was the best way to protect the patient, as well as it being within the remit of the Abortion Act 1967.^71^ What is similarly clear is that they did not work to support her in making a decision for herself, and it was only concerns about whether the pregnancy had progressed past the permitted gestation that resulted in the pregnancy continuing.

The second challenge highlighted by this case is that posed by gestational limits where capacity assessments are complex or disputed. As already mentioned, the impact of gestational limits on access to abortion is well established, particularly their disproportionate impact on people more likely to experience later pregnancy detection including people with disabilities. In circumstances where questions of legal capacity arise, the compression of time that gestational limits create in abortion-seeking is exacerbated. Capacity, in English law, is decision-specific. Under English law, a person aged 16 years and over is presumed to have the capacity to make their own decisions.^72^ In medical settings, this means that a person has an absolute right to choose a medical intervention or to refuse it. There are three main approaches to the assessment of capacity. The first one is the status approach, which accepts that some people may lack decision-making capacity because of their status, such as being under a specific age or having a disability. Another approach to capacity is outcome-based, where one’s capacity is judged based on the decision made. Finally, the functional approach places an emphasis on an individual’s decision-making capabilities. The Mental Capacity Act 2005 (MCA 2005) promotes people’s autonomy by rejecting outcome-based approaches to capacity and maintaining that people have the right to make decisions that may appear unwise.^73^ The MCA 2005 adopts primarily a functional approach to capacity, which means that to have capacity, a person must be able to understand the information about the decision^74^ that is given to them and must be able to retain that information^75^ even for a short amount of time,^76^ use the information to weigh up the decision^77^ and, finally, to communicate their decision in whatever way available.^78^ The MCA 2005 operates alongside several principles, including supported decision-making.^79^ This means the person should be actively supported to retain their mental capacity. In the context of learning disabilities, for instance, this means that information must be provided in an accessible way to enable the person to have the understanding necessary to make their decision.^80^ How the person communicates their decision must also be supported and respected.

Although the courts^81^ maintain that English law adopts a functional approach to capacity, the reality is that the test remains a combination of the status and the functional approaches to capacity. Thus, questioning people’s capacity will likely happen in one of two situations. When a person belongs to a group whose population tends to lack capacity, healthcare professionals are likely to question their capacity, and this group will include pregnant women with disabilities or women with mental ill-health.^82^ Secondly, the apparent irrationality of a person’s choice will likely lead to questioning their capacity, even though the perceived wisdom of a decision cannot determine the question of capacity. This can often be observed in cases where pregnant people refuse caesarean interventions.^83^ The realities surrounding mental capacity assessments place pregnant people, and especially pregnant disabled people, in a position where determining capacity can lead to delays. If one is already approaching the gestational limit for lawful abortion, those delays can have clear and grave implications for the pregnant person’s ability to exercise reproductive agency, supported—where needed and appropriate—in their decision-making.

### B. Lack of legal capacity

There will, of course, be cases where a pregnant person is deemed not to have capacity to decide whether to continue with pregnancy or not. In those cases, a process to enable the decision to be made on their behalf is undertaken and that, too, introduces delays although it does not (of course) have any impact on the applicability of the statutory gestational limits or the criminalization of abortion provision once those limits have been exceeded. Decisions as to abortion or continuation of pregnancy by persons without capacity are made based on the best interests principle.^84^ Best interests assessments consider a wide range of factors, and the list contained in section 4 of the MCA 2005 is not exhaustive and clinical consideration will only form part of the decision. While the views of all relevant persons will be taken into account, ascertaining past and present wishes and feelings of the person at the heart of the decision will be a paramount consideration, although it may not necessarily be decisive.^85^ The principles of the MCA 2005 further state that when deciding between different possible outcomes, the presumption should always be in favour of the least restrictive option,^86^ although it may not always be entirely clear what is ‘less restrictive’ in the context of pregnancy.

That this legal background further complicates and sometimes delays highly time-sensitive decisions surrounding continuation of pregnancy is illustrated by the case of *Re AB.*^87^ AB was a 24-year-old Nigerian woman who was adopted from birth by CD. Although AB did not have a formal diagnosis of mental disability, her IQ was in the range of 35–49, and her challenging behaviour and aggressive outbursts were managed by prescription medication, and she attended special needs schools throughout her life. In addition, her spoken English was difficult to understand by others. Following a visit to Nigeria in 2018, CD discovered that AB was pregnant. The relevant NHS Trust sought an abortion based on AB’s severe intellectual disability. Due to associated delays in proceedings, AB was already at 22 weeks’ gestation at the time of the hearing. AB’s mother was a devout Roman Catholic who strongly opposed the termination. She believed that AB would want the child and that she would find termination especially distressing,^88^ a view supported by the social worker.^89^ The relevant expert witnesses diagnosed AB with intellectual disability^90^ and suggested that AB might be suffering from psychosis.^91^ Expert witnesses were also concerned about the likelihood of postpartum psychosis.^92^ They became concerned about how AB would cope with ante-natal care and the possibility of a caesarean section, given the seriousness of the surgery.^93^ There was also a possibility of AB losing her home should she remain pregnant. The High Court criticized the delay in proceedings, considering the law’s imposed gestational limits,^94^ but granted an order for termination following expert witnesses’ statements.^95^ However, CD appealed the decision claiming that it was erroneous to assume AB would lose her home should she continue the pregnancy, arguing that anticipated grave risk to AB should be proven before granting termination, and arguing for further weight to be given to AB’s ‘wishes and feelings’ under the best interests assessment in light of AB’s Article 8(1) rights.^96^ This third ground of appeal proved particularly challenging and ultimately led to the reversal of the previous order for termination. King J in the Court of Appeal stated that the Court of Protection did not balance AB’s best interests properly by not ascribing a particular weight to AB’s wishes, including the wishes that were communicated to the Court by CD and AB’s social worker.^97^ AB’s fleeting feelings towards her doll, which she sometimes considered her baby, were also considered.^98^ King J concluded that ‘[t]he medical evidence alone, did not convincingly demonstrate the need for such profound intervention as a termination of a pregnancy’^99^ and noted that even though AB will not be able to keep her child once born, she ultimately wants it.^100^ Consequently, the best interests consideration of appeal resulted in the overturning of the Court of Protection’s judgment.^101^

Cherkassky criticizes the judgment handed down by King J, noting that the evidence of AB’s wishes and feelings were limited to the opinion expressed by CD and AB’s social worker alongside AB having some feelings towards a doll and questions whether these constituted sufficient evidence to outweigh other considerations in the best interests assessment.^102^ Furthermore, the decision has been criticized for setting aside an order for termination on the basis that ‘significant weight’ was not ascribed to wishes and feelings,^103^ especially considering the developments in this area requiring assessors to give particular weight to ascertaining wishes and feelings but understanding these will not always prevail.^104^

For our purposes, *Re AB* demonstrates the complexity of decisions relating to termination in situations where the pregnant person is not capacitous. Regardless of one’s views on the quality of argumentation or outcome of the case, *Re AB* illustrates very well how multi-faceted, disputed, and complicated the factors to be considered—as well as the weighting they should receive—are in such cases. Clearly, the delays that are endemic to and caused by capacity assessment and subsequent ‘best interests’ analysis—and thus to ‘consenting’ people with disabilities’ decision to access a particular health intervention—are always problematic, but they are especially challenging in the context of abortion law which imposes a time-based limitation on legality. Even if these processes do not result in someone falling outside of eligibility (because the gestational limit is exceeded), they can result in them needing to access care later in the 24-week period. This poses its own challenges. While abortion remains lawful up to 24 weeks gestation, it becomes less accessible as gestational age advances simply because of the limited availability of providers at later gestations and because a more limited range of abortion methods might be available. Although disabled women are not the only group of pregnant people who may be found not to have capacity and thus subjected to these processes of decision-making, it is foreseeable that they constitute a disproportionate portion of pregnant people in this situation and, therefore, its implications in the context of gestational limits combined with criminalization of abortion fall especially heavily on them.

### C. Models of service provision

The Royal College of Obstetricians and Gynaecologists (RCOG) reports that 77 per cent of all abortions funded by the NHS in England and Wales (through commissioning)^105^ are provided by three major independent providers.^106^ Only a small number of remaining abortions are performed through NHS Trusts. This suggests that most disabled people who present seeking abortion are likely to be seen, at least at first instance, by independent providers with limited resources, facilities, and time to ensure effective and appropriate provision of care in complex cases. RCOG notes that specialist care requires specialist commissioning, which is not as widely available and poses particular challenges due to delays caused by travel times, specialist needs, and particularly later gestations:

Later gestation cases need more specialist skills and resources. These services should be available within each region, or in the most complex circumstances nationally. Commissioners and providers should collaborate with regional MDTs [multidisciplinary teams] to ensure that provision for later gestation and complex cases is integrated within the regional and national framework. Commissioners and providers should work together to ensure that women are promptly referred onwards if a service cannot provide an abortion after a specific gestational age or by the woman’s preferred method.^107^

Across the commissioning landscape, there are ‘significant variations in the quality-of-service specifications’,^108^ while under-resourcing of abortion means ‘essential but more costly elements of the pathway, such as safeguarding and later gestation care, are not sustainable without commissioning change’^109^ (ie, within the independent provider sector) and difficult to access in the NHS (because of skill depletion as a result of most abortion care being provided outside of the NHS itself).^110^ It seems likely to us that the challenge to effectively provide quality abortion care for disabled women, as revealed here, is not solely attributable to the independent providers themselves, per se, but to the commissioning structure for abortion care, which depletes capacity to provide quality abortion care in complex cases across both the independent and NHS sectors, thereby exacerbating constraints imposed by the legal framework contained within the Abortion Act 1967.

Courts have previously suggested that every abortion care provider should have internal guidelines on how abortion care is provided to women experiencing mental ill-health,^111^ and scholars affirm the necessity of frameworks to support appropriate decision-making in such cases.^112^ Despite this, the existence or content of such policies cannot be confirmed through publicly accessible materials. Although speculative, this gives rise to the possibility of significant variations in complex care provision across providers and makes it difficult to ascertain whether existing arrangements learn from and adhere to international human rights law. On a more prosaic level, the opacity of how providers support disabled women in accessing abortion care means that they are unlikely to have sufficient information to make a choice about which provider is most likely to be able to meet their needs and respect their preferences regarding the termination of pregnancy, the method of abortion, and pre-and post-abortion care.

## IV. FACING REALITIES AND PROMOTING REPRODUCTIVE AGENCY OF WOMEN WITH DISABILITIES: FOSTERING HUMAN RIGHTS COMPLIANCE

It is clear, as we have argued thus far, that the law or, at the very least, abortion-related policy and regulation must promote and facilitate disabled pregnant people’s reproductive agency by developing appropriate guidelines and mechanisms for supporting them in accessing abortion on an equal basis with others. Although specific evidence on the impact of abortion laws and policies on disabled and pregnant people is lacking, we do know that there are significant difficulties in ensuring supported decision-making across various domains of complex healthcare. For instance, empirical work by Harding and Tascioglu^113^ suggests that people with disabilities are better supported in making day-to-day decisions about their lives than they are in making complex decisions regarding medical treatment. They argue that one of the biggest obstacles to providing adequate support in these contexts is our inability to either provide or understand what it means to provide information in an accessible manner to people with disabilities.^114^ This is notwithstanding the fact (as mentioned earlier) that Article 9 of the CRPD, related to accessibility, speaks to accessibility not only of physical spaces but also of language and information provision.^115^ This is further supported by the CRPD’s Article 21 on freedom of expression, which states that the provision of accessible information is a necessary aspect of informed consent. In other work, Furgalska has shown how disabled women’s reproductive agency is limited by the failure to provide them with relevant information about specific treatments, including sexual and reproductive health care.^116^ Thus, broadly conceived accessibility should be the pinnacle of supported decision-making in the context of meaningful abortion-care provision for disabled women.

### A. Accessibility of method and space

A broader understanding of accessibility, inclusive of both physical and nonphysical aspects, is crucial for providing accessible and high-quality abortion care that will bring English and Welsh abortion regulation and policy closer to international human rights compliance. With respect to physical spaces and treatment options, accessibility can be improved by providing a wider choice in abortion methods (including not assuming that medical abortion is appropriate or suitable for every early termination) and locations (including not assuming that every medical abortion should be conducted at home). Fundamentally, the key is ensuring that women have options regarding space and method, and crucially, that they are supported in making decisions among these options. Importantly, this is of significance to all people seeking abortion, not just women with disabilities or mental ill-health. As Parsons and Romanis put it:

It is the role of the HCP [health care professional] to advise the patient as to what the clinically appropriate options are, alongside a reporting of the benefits and drawbacks of each in as objective terms as possible. In the context of abortion, for example, it would be appropriate to explain what the patient will experience depending on the method chosen, allowing a patient with a serious aversion to blood, for example, to make an informed decision that may well result in them opting for a surgical rather than medical method.^117^

It is not difficult to imagine how aversion to blood or symptoms of mental illness characterized by hallucinations or delusions in relation to blood might render the medical abortion care pathway inappropriate in terms of supporting meaningful abortion access. On the contrary, surgical abortion may cause distress to disabled women who have a particular fear of medical settings; thus, it will become necessary to consider the ways in which changing the physical space might become a mode of support depending on the individual needs, preferences, and experiences of the individual woman.

The recommended method of abortion can also play a significant role in a Court’s decision on whether a pregnancy should be continued instead. In particular, there will be cases where the recommended method of abortion has been chosen without taking into account the pregnant woman’s disability or mental health needs. Instead, a particular abortion method might be deemed inappropriate because of assumptions made about the pregnant person’s disability. Unavailability of particular abortion methods that cater to individual needs in this context might ultimately lead to the denial of abortion care altogether. In this context, courts might decide in favour of continuation of pregnancy on the basis that a particular method of abortion is assumed to be more traumatic for her than continuation of the pregnancy rather than relying on disabled women’s preferences. This is amply illustrated by *Re SS*,^118^ which concerned an application to declare *SS* incapacitated to decide about abortion at 24 weeks gestation. *SS* was diagnosed with schizophrenia and was detained in a mental health hospital. In this case, the abortion care pathway proved to be the main obstacle in accessing abortion and making decisions about termination. The method proposed by experts for termination included foeticide followed by induction of labour. Experts, in this case, believed that this method of abortion would be no less traumatic than a normal birth followed by removal of the child for adoption. Ultimately, it was found that continuing with the pregnancy would have been less confusing for *SS* than the proposed medically appropriate pathway.^119^ Wall J held that it was necessary for each psychiatric hospital to have procedures in place that would enable speedy decision-making in such future cases to ensure that abortion can be carried out at the earliest opportunity. *Re SS* provides a good context to demonstrate what might change if the rights contained in the CRPD were better incorporated into decision-making. The right to accessibility under Article 9 would require the Court to consider all available care pathways before deciding that continuing with pregnancy was less traumatic or confusing and thus preferred. This would change not only the sense of the options available and appropriate for termination but also the calibration of the Court’s understanding of ‘trauma’. In previous works, scholars like Cherkassky^120^ and Hale^121^ suggested that the trauma of [unwanted] childbirth and subsequent loss of a child (being taken away by relevant authorities) should be given utmost consideration in abortion-related decisions in disabled women.

### B. Accessible information provision

As already noted, it is equally important that information about abortion is made accessible to disabled pregnant people. Providing accessible information is necessary for obtaining informed consent and minimizing instances of force and coercion.^122^ This could be achieved on both macro and micro scales. The macro-scale would require the state to commission the creation of appropriate accessible information on abortion decision-making, abortion care pathways and evidence-based knowledge on abortion more generally. The creation of such resources should be overseen by relevant governmental departments such as the Department of Health and Social Care. This is instrumental for safeguarding information, which is necessary in the context of abortion. Evidence suggests that knowledge of abortion law and abortion care pathways is poor among the general population.^123^ In addition, the NHS had been subject to a number of scandals in 2014 and 2023 exposing that the NHS unknowingly awarded abortion counselling contracts to anti-abortion groups, which were being promoted to patients on the NHS websites.^124^ Furthermore, in 2023, Google was exposed for directing pregnant women to services run by anti-abortion groups and was accused of displaying deceptive adverts in relation to abortion information in the UK.^125^ Considering the problems surrounding general access to information about abortion, it is imperative that accessible and reliable information on abortion exists that is safeguarded by the state. On a micro level, however, it would be useful to consider training healthcare providers to provide accessible information on abortion directly to individuals face-to-face.

### C. Ensuring human rights compliance

Whether or not abortion is the best course of action should be decided through supported decision-making, where different options are explained in an accessible manner that adequately supports the unique needs of individual pregnant people. It is, therefore, imperative that gestational age or common abortion care pathways are not the only considerations for choosing the best course of action, and that limitations in provisions of abortion should not be treated as automatic justifications for denying abortion care. The individual’s needs must pave the way for decision-making in this context (in accordance with the ethos of the CRPD), and adjustments should be made to ensure that abortion remains an accessible option.

Developing a framework for supported decision-making rooted in human rights affirming care and, in particular, the CRPD, would bring the Abortion Act 1967 somewhat closer to compliance with human rights obligations. However, the most meaningful form of supporting disabled women’s reproductive agency in relation to abortion access would be through engaging in law reform framed through the lens of rights-based approaches supported by existing evidence, such as those provided by the WHO in their *Abortion Care* guidelines. At the very least, the law reform would need to reconsider the appropriateness of gestational limits, as these pose particular challenges for disabled women. In addition, the policy agenda should focus on abortion care pathways and greater flexibility in choosing the methods of abortion to support access for disabled women. Finally, the state must work with providers towards implementation of accessible abortion information as a matter of urgency.

## V. CONCLUSIONS

As we have shown, international human rights law and, in particular, the CRPD, can inform how meaningful support is provided to disabled pregnant people who are pregnant and considering abortion or, having made the decision not to continue with pregnancy, are accessing abortion care. The primary goal of the CRPD has always been and remains support for equal enjoyment of the rights of disabled people. Thus, understanding the CRPD as a set of building blocks and tools for supporting reproductive agency is long overdue.

Critically, law-making in this area should be evidence-based.^126^ The experiences of people with disabilities in this context are largely invisible in the existing, empirical literature. This evidence gap can be addressed through robust, interdisciplinary, and empirical research to understand how the current legal regime governing abortion access for people with disabilities impacts their experience; how (and if) disabled, pregnant women and girls are currently supported in accessing abortion; and whether the support and decision-making provided comply with international human rights standards. Such research should adequately represent a range of disabilities, including mental ill-health, to ensure that appropriate tailor-made guidelines for support are available.

Even in advance of this specific research, however, international human rights law and public health evidence about the *general* impacts of abortion law and policy on health and human rights points towards the urgent need for reform. This includes formal legal changes relating, for example, to gestational age limits. In the absence of legislative reform, positive change can be introduced in practice, particularly by developing rights-affirming protocols for abortion care provision and access, and appropriately resourcing and training abortion providers to provide rights-based abortion care in ‘complex cases’.

Our analysis of the current legal and policy framework in England and Wales demonstrates that pregnant disabled people are unlikely to receive the support that rights-based approaches tell us is required to ensure effective exercise of rights relating to abortion. Disabled and pregnant women are more likely to be subjected to legal tests and procedures, like mental capacity assessments, ‘best interests’ assessments and court proceedings, which further convolute and delay decision-making processes in abortion care. This begs the question of whether disabled pregnant people are truly treated as equal before the law when evidence-based supported decision-making mechanisms tailored to abortion and pregnancy-related decisions are under-researched and, thus, likely not utilized in practice.

## Footnotes

1 Throughout this article, we use the terms women, girls, pregnant women (and girls), pregnant people, and people interchangeably to include all those with the capacity for pregnancy.

2 See eg, UN Committee on the Rights of Persons with Disabilities and UN Committee on the Elimination of Violence Against Women, ‘Guaranteeing sexual and reproductive health and rights for all women, in particular women with disabilities’ (29 August 2018) <https://www.ohchr.org/sites/default/files/Documents/HRBodies/CRPD/Statements/GuaranteeingSexualReproductiveHealth.DOCX> accessed 10 January 2024.

3 Some scholars have begun to consider disabled women’s rights in light of CRPD or in the context of disability more broadly, see eg, Charles Ngwena, ‘Reproductive Autonomy of Women and Girls under the Convention on the Rights of Persons with Disabilities’ (2017) 140 Gynecology & Obstetrics 128; Zoe L Tongue, ‘Human Rights and Foetal Impairment Grounds for Abortion: *Crowter v Secretary of State for Health and Social Care* [2022] EWCA Civ 1559’ [2023] Medical Law International 297; Lisa Cherkassky, ‘Re: AB (Termination of Pregnancy) [2019] EWCA CIV 1215: “Wishes and Feelings” Under the Mental Capacity Act 2005’ (2020) 28 Medical Law Review 605; Joanna M Manning, ‘“Turning Up the Volume”: Increasing Respect for Wishes and Feelings of Women with Intellectual Impairment in Decisions about their Reproductive Rights in England and New Zealand’ [2021] Medical Law International 243.

4 World Health Organization, *Abortion Care Guideline: 2022* (WHO 2022) 106.

5 See, for instance, the work of disabled scholar Robyn M Powell, ‘Disability and Reproductive Justice’ (2022) 170 University of Pennsylvania Law Review 1851. In this article, Powell discusses the historical and current reproductive oppression of people with disabilities, noting how race, sexuality, and gender can further exacerbate experiences of reproductive injustice, denial of choice and, thus, coercion.

6 Nina E Ross and others, ‘‘Reproductive Decision-Making Capacity in Women with Psychiatric Illness: A Systematic Review’ (2022) 63 Journal of the Academy of Consultation-Liaison Psychiatry 61, 62; Powell ibid.

7 Annika F Petersen, ‘Accessing Late-Term Abortion Following Sexual Assault: Looking Inside the Danish Legal Black Box’ (2022) 40 Nordic Journal of Human Rights 389, 403.

8 For historical context on disability, reproduction, and eugenics, see Claudia Malacrida, ‘Mothering and Disability: From Eugenics to Newgenics’ in Nick Watson and Simo Vehmas (eds) *Routledge Handbook of Disability Studies* (2nd edn, Routledge 2020).

9 In England and Wales, these discussions are particularly visible following the judgment in *Crowter v Secretary of State for Health and Social Care* [2021] EWHC 2536, see eg, discussions in Sally Sheldon, ‘Beyond the Tram Lines: Disability, Discrimination, Reproductive Rights and Anachronistic Abortion Law’ (2023) XX Oxford Journal of Legal Studies 1 on limits of the Abortion Act 1967 and the ground of disability, as well as for pointing out limitations in recent debates; Kavana Ramaswamy, ‘Legality of Disability Selective Abortion: Discrimination and Privacy Laws under the ECHR’ (2023) 4 European Human Rights Law Review 333; Zoe Tongue, ‘Crowter v Secretary of State for Health and Social Care [2021] EWHC 2536: Discrimination, Disability and Access to Abortion’ (2022) 30 Medical Law Review 177; for broader discussions see eg, Ottavio Quirico and others, ‘Right to Life v Right to Health? Disability and Selective Abortion’ in Ottavio Quirico (ed), *Inclusive Sustainability: Harmonising Disability Law and Policy* (Springer 2022); Tom Shakespeare, ‘Choices and Rights: Eugenics, Genetics and Disability Equality’ (1998) 13 *Disability & Society* 665; Keith Sharp and Sarah Earle, ‘Feminism, Abortion and Disability: Irreconcilable Differences?’ (2002) 17 Disability & Society 137; A Ash, ‘Prenatal Diagnosis and Selective Abortion: A Challenge to Practice and Policy’ (1999) 89 American Journal of Public Health 1649.

10 See commentary from Zoe Louise Tongue, ‘Human Rights and Foetal Impairment Grounds for Abortion: *Crowter v Secretary of State for Health and Social Care* [2022] EWCA Civ 1559’ [2023] Medical Law International 297.

11 This is particularly apparent in anti-abortion activism, see eg, ADF International ‘Submission to the CRPD Committee on General Comment No.6 on Article 5 of the CRPD—Equality and Non-Discrimination’ (30 November 2017), paras 2 and 3 asking for explicit recognition that art 10 (right to life) applies to ‘unborn persons with disabilities’; Ordo Iuris, ‘Danger for the Protection of Life and Family: Reports to the UN’ (17 August 2018) <https://en.ordoiuris.pl/life-protection/danger-protection-life-and-family-reports-un> accessed 24 January 2024. Here, Ordo Iuris argues that the CRPD prohibits eugenic abortion. Similar arguments were put forward in the case concerning legality of abortion on the grounds of fatal foetal anomaly, Wyrok Trybunalu Konstytucyjnego z dnia 22 pazdziernika 2020 roku, K 1/20 Planowanie rodziny, ochrona płodu ludzkiego i warunki dopuszczalności przerywania ciąży, K 1/20, orzeczenie 4/A/2021: Constitutional Tribunal, Decision, 22 October 2020, Family Planning, Protection of the Foetus and Conditions for the Admissibility of Abortion, K 1/20, Judgment 4/A/2021.

12 ibid.

13 Committee on the Rights of Persons with Disabilities and Committee on the Elimination of Violence against Women, ‘Guaranteeing Sexual and Reproductive Health and Rights for all Women, in Particular Women with Disabilities’ (29 August 2018) <https://www.ohchr.org/sites/default/files/Documents/HRBodies/CRPD/Statements/GuaranteeingSexualReproductiveHealth.DOCX> accessed 5 December 2024.

14 UN CRPD, art 8.

15 The Offences Against the Person Act 1861 contains two specific abortion offences provisions. The first one relates to ‘unlawful procurement of miscarriage’ contained in s 58 of the Act, and the second relates to supplying a substance or procuring an instrument that is intended to procure a miscarriage. The criminalization of abortion in the UK remains an important issue as recently highlighted in the case of *R v Foster* (Crown Court Stoke on Trent, 12 June 2023) <https://www.judiciary.uk/wp-content/uploads/2023/06/R-v.-Foster-sentencing-remarks-12.6.23.pdf> accessed 5 December 2024; see also Elizabeth C Romanis, ‘R v Foster: Exemplifying the Urgency of the Decriminalisation of Abortion’ (2023) 31 Medical Law Review 606; See Sally Sheldon and others, *The Abortion Act 1967: A Biography of a UK Law* (CUP 2023) for a comprehensive historical, political, and legal account of developments in this area.

16 Abortion Act 1967, s 1.

17 ibid s 4.

18 ibid s 1(1)(a).

19 ibid s 1(1)(b).

20 ibid s 1(1)(c).

21 ibid.

22 Piers Gooding, *A New Era for Mental Health Law and Policy: Supported Decision-Making and the UN Convention on the Rights of Persons with Disabilities* (CUP 2017) 119. Gooding first used the idea of building blocks in relation to CRPD to highlight the indivisibility and interdependent character of rights, which within the CRPD include both civil, political, and socio-economic rights.

23 For a full, comprehensive outline of international human rights standards related to abortion see WHO, ‘Web Annex A. Key International Human Rights Standards on Abortion’ in *WHO*, *Abortion Care Guideline*: *2022* (World Health Organisation 2022).

24 UN Committee on Economic, Social and Cultural Rights, ‘General Comment No 22 on the Right to Sexual and Reproductive Health’ (art 12 of the International Covenant on Economic, Social and Cultural Rights) (2 May 2016), UN Doc E/C/12/GC/22, para 49; UN Committee on the Rights of the Child, ‘General Comment No 4 on Adolescent Health and Development’ (1 July 2003), UN Doc CRC/GC/2003/4, paras 6, 9, 24, 30–33.

25 UNHRC, ‘General Comment No 36 on Article 6 of the International Covenant on Civil and Political Rights, on the Right to Life’ (30 October 2018), UN Doc CCPR/C/GC/36, para 8.

26 ibid; UN Committee on Economic, Social and Cultural Rights (n 24) para 28.

27 UN Convention for the Elimination of All Forms of Discrimination against Women, ‘General Recommendation No 24: Article 12 of the Convention (Women and Health)’ (20 August 1999) UN Doc A/54/38/Rev.1, Ch I, para 11; UNHRC, ‘General Comment No 28: Article 3 (The Equality of Rights between Men and Women)’ (2000) UN Doc CCPR/C/21/Rev.1/Add.10.

28 UN Committee on the Economic, Social and Cultural Rights (n 24), paras 20 and 34; UN Convention for the Elimination of All Forms of Discrimination against Women, ‘General Recommendation 35 on Gender-based Violence against Women, updating General Recommendation No 19’ (26 July 2017), UN Doc CEDAW/C/GC/35, para 18; UN Convention for the Elimination of All Forms of Discrimination against Women General Recommendation 33 on Women’s Access to Justice (3 August 2015), UN Doc CEDAW/C/GC/33, para 51(I); UN Committee on the Rights of the Child, General Comment 20 on the Implementation of the Rights of the Child during Adolescence (6 December 2016), UN Doc CRC/C/GC/20, para 60; Working Group on the Issue of Discrimination Against Women in Law and in Practice (8 April 2016), UN Doc A/HRC/32/44, paras 82 and 107; UNHRC (n 25) para 8.

29 UNHRC (n 26) para 8.

30 WHO (n 4). For the purposes of transparency, we confirm that the authors of this paper were commissioned to design and deliver research that developed the evidence base on abortion law and policy that informed the recommendations adopted in this *Guideline*.

31 ibid 24; see also, Fiona de Londras and others, ‘The Impact of Criminalisation on Abortion-related Outcomes: A Synthesis of Legal and Health Evidence’ (2022) 7 BMJ Global Health e010409.

32 WHO (n 4) 26–27; see also Fiona de Londras and others, ‘The Impact of ‘Grounds’ on Abortion-related Outcomes: A Synthesis of Legal and Health Evidence’ (2022) 936 BMC Public Health 13247.

33 WHO (n 4) 28–29.

34 ibid, see ch 3; see also Fiona de Londras and others, ‘The Impact of Third-party Authorisation Requirements on Abortion-related Outcomes: A Synthesis of Legal and Health Evidence’ [2023] BMC Public Health 2065; Fiona de Londras and others, ‘The Impact of ‘Conscientious Objection’ on Abortion-related Outcomes: A Synthesis of Legal and Health Evidence’ (2023) 129 Health Policy 104716; Fiona de Londras and others, ‘The Impact of Mandatory Waiting Periods on Abortion-related Outcomes’ [2022] BMC Public Health 1232; Fiona de Londras and others, ‘The Impact of Provider Restrictions on Abortion-related Outcomes: A Synthesis of Legal and Health Evidence’ (2022) 19 Reproductive Health 95.

35 WHO (n 4) 106.

36 Ross (n 6) 64.

37 ibid.

38 ibid 61.

39 WHO and United Nations, *Mental Health, Human Rights and Legislation: Guidance and Practice* (Geneva 2023) para 1.5.5.

40 See WHO, ‘QualityRights Materials for Training, Guidance and Transformation’ <https://www.who.int/publications/i/item/who-qualityrights-guidance-and-training-tools> accessed 1 February 2024; see also Michelle Funk and others, ‘Strategies to Achieve a Rights-Based Approach through WHO QualityRights’ in Michael Ashley Stein and others (eds), *Mental Health, Legal Capacity and Human Rights* (CUP 2021); Richard M Duffy, ‘Responses to the World Health Organizations QualityRights Initiative’ in Brendan D Kelly and Mary Donnelly (eds), *Routledge Handbook of Mental Health Law* (Routledge 2024).

41 UN Committee on the Rights of Persons with Disabilities, ‘Concluding Observations on the Initial Report of the United Kingdom of Great Britain and Northern Ireland’ 3 October 2017, UN Doc CRPD/C/GBR/CO/1, paras 12–13.

42 See ADF International and Ordo Iuris (n 11).

43 See de Londras and others (n 32).

44 CRPD, art 12(1).

45 ibid art 12(2).

46 ibid art 12(3).

47 ibid art 12(4).

48 ibid art 17.

49 Penelope Weller, *New Law and Ethics in Mental Health Advance Directives: The Convention on the Rights of Persons with Disabilities and the Right to Choose* (Routledge 2013) 39; UN Committee on Economic, Social and Cultural Rights (n 24) para 9 and UNHRC (n 25) para 8 require the states to provide free accessible information about abortion. Special Rapporteur on the right of everyone to the enjoyment of the highest attainable standard of physical and mental health. Report to the General Assembly. United Nations; 2009 (A/64/272) para 13 obliges the States to ensure informed consent is documented without coercion or undue influence; para 15 ensures respect for a person refusing to receive information about interventions; para 7 informed consent is a right that must be legally safeguarded.

50 Gooding (n 22) 118.

51 Amita Dhanda, ‘Constructing a New Human Rights Lexicon: Convention on the Rights of Persons with Disabilities’ (2008) 5 Sur-International Journal of Human Rights 45.

52 Gooding (n 22) 119.

53 CRPD, art 9.

54 Andrea Broderick, ‘Of Rights and Obligations: The Birth of Accessibility’ (2020) 24 International Journal of Human Rights 393.

55 de Londras and others (n 32); Gestational limits are likely to pose significant problems in many jurisdictions. In an Argentinian case concerning a 20-year-old woman with intellectual disability, who became pregnant as a result of rape, was denied abortion due to gestational limits. The UNHRC found that denying her abortion in this circumstance violated her human rights and that the victim’s status as a young woman with a disability made this violation even more serious. This suggests that human rights place a greater duty on protecting the rights of disabled women due to their inherent vulnerability. See *VDA v Argentina* CCPR/C/101/D/1608/2007.

56 de Londras and others (n 31).

57 de Londras and others, ‘The impact of third-party authorisation requirements’ (n 34).

58 See Jean O’Hara, ‘Pregnancy in a Severely Mentally Handicapped Adult’ (1989) 15 Journal of Medical Ethics 197.

59 Petersen (n 7) 403.

60 O’Hara (n 58) 197.

61 It is important to note that this case reports dates almost two decades prior to the enactment of the Mental Capacity Act 2005. Nonetheless, it serves as an important example of the problematic nature of gestational limits.

62 O’Hara (n 58) 197.

63 ibid.

64 ibid 198.

65 ibid.

66 ibid.

67 ibid.

68 ibid.

69 ibid.

70 ibid.

71 ibid.

72 Mental Capacity Act 2005, s 1(2).

73 ibid s1(4).

74 ibid s 3(1)(a).

75 ibid s 3(1)(b).

76 ibid s 3(3).

77 ibid s 3(1)(c).

78 ibid s 3(1)(d).

79 ibid s1(3).

80 ibid s 3(2).

81 In the *A Local Authority v JB* [2021] UKSC 35, paras 57–62, Lord Stephens stated that the law rejects status-outcome approaches to capacity in favour of the functional approach.

82 s 2(1) of the MCA 2005 states that: ‘a person lacks capacity in relation to a matter if at the material time he is unable to make a decision for himself in relation to the matter because of an impairment of, or a disturbance in the functioning of, the mind or brain’. This means that a person with disability does not automatically lack mental capacity as long as they satisfy the functional test contained in s 3 of the Act; see also Sara Fovargue, ‘Anticipating Issues with Capacitous Pregnant Women: United Lincolnshire NHS Hospitals Trust v CD [2019] EWCOP 24 and Guys and St Thomas’ NHS Foundation Trust (GSTT) and South London and Maudsley NHS Foundation Trust (SLAM) v R [2020] EWCOP 4’ (2020) 28 Medical Law Review 781.

83 See Fovargue ibid; Mary Walstead, ‘Was from his Mother’s Womb Ultimately Ripp’d (Macbeth Act 5, Scene 8): Court Ordered Caesarean Births’ (2015) International Survey of Family Law 33; Neha A Deshpande and Corrina M Oxford, ‘Management of Pregnant Patients Who Refuse Medically Indicated Cesarean Delivery’ (2012) 5 Medical Reviews in Obstetrics & Gynecology 144.

84 Cherkassky (n 3).

85
*In Aintree University Hospitals NHS Foundation v James* [2013] UKSC 67, Lady Hale at para 45 affirmed that ‘the purpose of best interests test is to consider matters from the patient’s point of view. That is not to say that his wishes must prevail, any more than those of a fully capable patient must prevail’.

86 MCA 2005, s 1(6).

87
*Re AB (Termination of Pregnancy)* [2019] EWCOP 26.

88 ibid para 26.

89 ibid.

90 ibid para 7 (per Professor X, a consultant psychiatrist).

91 ibid para 15 (Dr N, a perinatal psychiatrist).

92 ibid para 24 (Dr N).

93 ibid.

94 ibid para 5 (Lieven J).

95 ibid paras 62 and 63 (Lieven J).

96 ibid para 32.

97 ibid para 65.

98 ibid para 25.

99 ibid para 73.

100 ibid para 72.

101 ibid para 80.

102 Cherkassky (n 3) 611.

103 ibid.

104
*Aintree* (n 95); *Wye Valley NHS Trust v B* [2015] EWCOP 60. See also *Re SB (A Patient)* (Capacity to Consent to Termination) [2013] EWHC 1417.

105 Royal College of Obstetricians and Gynecologists (RCOG), *National Service Specifications for NHS Abortion Care* (RCOG 2022) 5.

106 ibid; British Pregnancy Advisory Service, National Unplanned Pregnancy Advisory Service, and MSI Reproductive Choices.

107 ibid 22.

108 ibid 5.

109 ibid.

110 ibid 22.

111
*Re SS (An Adult: Medical Treatment)* [2002] 1 FCR 73, in this judgment Wall J suggested that each hospital/provider should have internal guidelines on how specialised abortion care is provided to pregnant persons with mental-ill health.

112 Christopher Johnston and Sophia Roper, *Medical Treatment: Decisions and the Law* (Bloomsbury 2022) paras 9.23–9.25.

113 Rosie Harding and Ezgi Tasciouglu, *Everyday Decisions Project Report: Supporting Legal Capacity through Care, Support and Empowerment* (Birmingham Law School 2017) 30–33..

114 ibid 30.

115 Jaime Lindsey, *Reimagining the Court of Protection: Access to Justice in Mental Capacity Law* (CUP 2022) 196.

116 Magdalena Furgalska, ‘Informed Consent is a Bit of a Joke to Me: Lived Experiences of Insight, Coercion and Capabilities in Mental Health Care Settings’ (2023) 19 International Journal of Law in Context 456, see discussion at 467–469.

117 Jordan Parsons and Elizabeth Chloe Romanis, *Early Medical Abortion, Equality of Access and the Telemedical Imperative* (OUP 2021) 110.

118
*Re SS (Medical Treatment, Late Termination)* [2002] 1 FLR 445.

119 ibid.

120 Cherkassky (n 3) 607.

121 Brenda Hale, *Mental Health Law* (Sweet & Maxwell 2017) para 10.104.

122 UN Committee on the Rights of Persons with Disabilities, General Comment No 3: art 6: Women and girls with disabilities, (2016) UN DocCRPD/C/GC/3; Special Rapporteur on the right of everyone to the enjoyment of the highest attainable standard of physical and mental health, Report (2004) UN DocE/CN.4/2004/49; Special Rapporteur on the right of everyone to the enjoyment of the highest attainable standard of physical and mental health, Interim Report to the General Assembly (2011) UN DocA/66/254.

123 See discussion in Sally Sheldon and Kaye Wellings, *Decriminalisation Abortion in the UK: What Would It Mean?* (Bristol University Press 2020) 3–4.

124 See eg, Sophia S Galer ‘The NHS Awarded a Counselling Contract to a Group Set Up to Stop Abortions’ (Vice, 13 June 2023) <https://www.vice.com/en/article/ak334k/nhs-seen-charity-abortion> accessed 28 January 2024; Sophia S Galer, ‘The NHS Website Promoted 7 Counselling Centres Linked to Anti-Abortion Groups’ (Vice, 16th May 2023) <https://www.vice.com/en/article/wxjnqx/nhs-website-crisis-pregnancy-centres> accessed 28 January 2024; see also Eleanor Layhe and Divya Talwar, ‘Abortion UK: Women ‘Manipulated’ in Crisis Pregnancy Advice Centres’ (*BBC News*, 27 February 2023) <https://www.bbc.co.uk/news/uk-64751800> accessed 28 January 2024.

125 Efe Udin, ‘Google was Caught Displaying Deceptive Ads in the UK’ (Gizchina, 26 February 2023) <https://www.gizchina.com/2023/02/26/google-was-caught-displaying-deceptive-ads-in-the-u-k/> accessed 28 January 2024.

126 Lucia B Pizzarossa and Patty Skuster, ‘Towards Human Rights and Evidence-Based Legal Frameworks for (Self-Managed) Abortion: A Review of the Last Decade of Legal Reform’ (2021) 23 Health and Human Rights Journal 199.

